# Tubal infertility and pelvic adhesion increase risk of heterotopic pregnancy after in vitro fertilization: A retrospective study

**DOI:** 10.1097/MD.0000000000023250

**Published:** 2020-11-13

**Authors:** Ruyu Pi, Yu Liu, Xia Zhao, Ping Liu, Xiaorong Qi

**Affiliations:** Department of Gynecology and Obstetrics, Development and Related Disease of Women and Children Key Laboratory of Sichuan Province, Key Laboratory of Birth Defects and Related Diseases of Women and Children, Ministry of Education, West China Second Hospital, Sichuan University, Chengdu, Sichuan Province, People's Republic of China.

**Keywords:** assisted reproductive technologies, heterotopic pregnancy, infertility

## Abstract

To analyze risk factors associated with heterotopic pregnancy and the uterine pregnant outcome of those patients after surgery.

We retrospectively analyzed 22 patients diagnosed as HP after in vitro fertilization (IVF) between January 2015 and December 2018.

HP was diagnosed at gestation age of 55.4 ± 11.8 days. HP were presented as irregular vaginal bleeding, abdominal pain, and sometimes no symptoms. 81.8% of ectopic lesion in HP occurred at fallopian tubes, especially ampullary; cornual pregnancy takes up 13.6%. Compared with clinical intrauterine pregnancy (IUP), IVF with tubal infertility factors had higher risks of HP (OR 4.185, 95% CI 1.080– 16.217); IVF with pelvic adhesion also had higher risks of HP (OR 5.552 95% CI 1.677–18.382); IVF with more than 2 embryos transferred increased risks of HP (OR 23.253, 95% CI 1.804–299.767). The abortion rates of surgery-treated HP and IUP after IVF were 27.8% versus 10.3% (*P* = .042).

These results demonstrate IVF with tubal infertility, pelvic adhesion or multiembryos transfer are risk factors of HP. Furthermore, surgery could induce abortion.

## 1 Introduction

Heterotopic pregnancy (HP) is defined as the coexistence of an intrauterine pregnancy and an ectopic pregnancy (EP). The incidence of HP is 1 in 30,000 among spontaneous pregnancies.^[1]^ With widespread application of assisted reproductive technologies (ART), the incidence of HP has risen to about 0.09% to 1.0%.^[2,3]^ HP has risk of EP rupture, which may cause life-threatening complications including hypovolemic shock and maternal death. The diagnosis of HP at the early stage is vital but difficult. The initial β-human chorionic gonadotropin (hCG) level and hCG rise are less predictive of HP.^[4]^ And EP lesion might be too small to be detected on transvaginal sonography (TVS) at early stage or be ignored by clinicians who found a visible intrauterine sac. It is worthy to be familiar with clinical characteristics and risk factors of HP after in vitro fertilization (IVF).

The primary treatment goal of HP is to remove the ectopic sac while preserving the normal intrauterine pregnancy and leading to live births. Treatments include laparotomy or laparoscopic surgery, ultrasound-guided local injection of potassium chloride (KCL), methotrexate (MTX), or aspiration of the gestational sac.^[1,2,5–9]^ Laparoscopic surgery is used in most HP cases, but the pregnancy outcomes of HP after laparoscopic surgery are uncertain.

The objectives of our retrospective study were to assess risk factors for HP after IVF, and to compare pregnancy outcomes of surgery-treated HP with clinical intrauterine-only pregnancy, by analyzing HP data from West China Second Hospital between 2015 and 2018.

## 2 Methods and statistics

We retrospectively analyzed all the patients diagnosed as “heterotopic pregnancy” between January 2015 and December 2018 at West China Second Hospital. A total of 22 patients with diagnosed HP after IVF were included in this study. This study was approved by the ethics committee of West China Second Hospital (No. 2019-080).

For this study, we selected procedures performed in our assisted reproductive center from January 2015 to December 2018, which resulted in clinical intrauterine pregnancies (clinical IUP), intrauterine-only pregnancies (only-IUP), or HP. A clinical IUP was defined as 1 or more gestational sacs were in the uterus confirmed by ultrasound. An IUP-only was defined as 1 or more gestational sacs that were visible by ultrasound and located solely with the uterus. HP was defined as at least one gestational sac in the uterus, and one or more gestational sacs outside the uterus. Of the 3892 procedures performed in our hospital and confirmed as clinical IUP between 2015 and 2018, 3749 cycles were only-IUP and 22 cycles were HP.

We reviewed the clinical characteristics of HP, including maternal age, gestational age, previous ectopic pregnancies, previous abortions, previous live births, pelvic surgery history, whether with hypovolemic shock, EP location, whether EP lesion ruptured. Gestational age (GA) was calculated as date of diagnosis minus date of oocyte retrieval and fertilization, plus 2 weeks. When the date of the oocyte retrieval was unknown, or when frozen embryos were transferred, we calculated GA as the date of diagnosis minus the date of embryo transferred, plus 2 weeks. Pelvic surgery included salpingectomy, salpingostomy and fimbrioplasty, laparoscopic adhesionlysis, ovarian cyst excision, appendectomy. We also analyzed the pregnancy and delivery outcomes of HP and clinical IUP. These included live birth (preterm delivery or term delivery), abortion, pregnancy failure. Live birth was defined as the delivery of 1 or more infants with any signs of life. Preterm delivery was defined as GA <37 weeks; term delivery was defined as GA ≥37 weeks. Abortion was defined as a pregnancy ending in the spontaneous or an operative procedure loss of the embryo or fetus before 20 weeks of gestation. Pregnancy failure was defined as fetus stopped development after 20 weeks of gestation.

We evaluated IVF-associated factors and events, including type of IVF cycle (fresh or frozen-thawed), infertility factor, number of transferred embryos, stage of transferred embryos, and ovarian hyperstimulation syndrome (OHSS). We focused on 2 infertility factors (tubal infertility, pelvic adhesion). Tubal infertility included tubal obstruction, adhesion of fallopian, salpingitis or after salpingectomy caused infertility. Pelvic adhesion was defined as who were diagnosed as pelvic adhesion via laparoscopic examination.

To assess risk factors of HP after IVF, we randomly selected 125 intrauterine-only pregnancies and compared with heterotopic pregnancy. We analyzed potential risk factors: age (<30 years old and ≥30 years old), previous ectopic pregnancies, previous abortions, previous live births, pelvic surgery history, type of cycle (fresh or frozen-thawed), infertility factors, number of transferred embryos, stage of transferred embryos.

To assess the impact of laparoscopic treatment on HP outcomes, we compared the ratio of live birth and abortion between only-IUP and HP. We excluded 4 cases of HP, which were terminated gestation by curettage during HP surgery.

Differences between HP and IUP were analyzed by Chi-square tests at a significance level of *P* < .05. We used log-binomial regression models with odds risk (OR) and 95% confidence intervals (CI) for the association between predicted risk factors and heterotopic pregnancy. Data were analyzed via SPSS 25.0.

## 3 Results

In our study, HP generally occurred in female at age of 29.0 ± 3.4 years old (range, 21 years old to 36 years old), and were diagnosed at gestation age of 56.0 ± 11.5 days (range, 40 days to 79 days). The clinical characteristics of HP were listed in Table 1.

**Table 1:** Summary of characteristics and uterine pregnancy outcomes of HP.

Clinical characteristics of HP	Patients with HP (*n* = 22)
Age, mean ± SD (range), years	29.0 ± 3.4 (21–36)
GA at diagnosis, mean ± SD (range), days	56.0 ± 11.5 (40–79)
Symptoms of HP, *n* (%)	
Vaginal bleeding	12 (54.5)
Abdominal pain	9 (40.9)
Other symptoms (nausea, vomiting, dizziness or fatigue, etc.)	6 (27.3)
No symptoms	5 (22.7)
Rupture of EP, *n* (%)	2 (9.1)
Location of EP, *n* (%)	
Fallopian tube	18 (81.8)
Ampullary	7 (31.8)
Isthmic	6 (27.3)
Tubal stump	1 (4.5)
Interstitial	4 (18.2)
Uterine corn	3 (13.6)
Ovary	1 (4.0)
Uterine pregnancy outcome,	
Curettage during HP surgery, *n* (%)	4 (18.2)
Abortion or pregnancy failure, *n* (%)	5 (22.7)
Live birth delivery, *n* (%)	13 (59.1)
At term delivery, *n*	7 (53.8)
Preterm delivery, *n*	6 (46.2)

The symptoms of HP were reported as irregular vaginal bleeding, abdominal pain, and sometimes dizzy or nausea in cases (listed on Table 1). Twelve patients (54.5%) had vaginal bleeding, and 9 patients (40.9%) had abdominal pain, but 5 patients (22.7%) had no symptoms and were found by routine ultrasound examination. Two patients were diagnosed with rupture of tubal EP, fortunately with no hypovolemic shock. Most of ectopic lesion in HP occurred at fallopian tubes, taking up 81.8% (18/22); and 18.2% (4/22) of ectopic lesion occurred at interstitial fallopian. And cornual EP takes up 13.6% (3/22) of HP after IVF. HP after IVF could occasionally happen at tubal stump or at ovary (the distribution of EP location was shown in Table 1). We reviewed previous ectopic pregnancy, previous abortion and pelvic surgery history in HP (listed in Table 2). 68.2% (15/22) of HP had no ectopic pregnancy before; and 59.1% (13/22) of HP had no abortion before. 50.0% (11/22) of HP experienced tubal surgery, including salpingectomy, salpingostomy, and fimbrioplasty. All HP received laparoscopic surgery and 4 patients had uterine curettage during HP surgery because of uterine embryo poor development, pregnancy outcomes of the remaining 18 HP were 13 patients (59.1%) had live birth delivery (7 at term and 6 preterm); 5 patients (22.7%) had abortion or pregnancy failure (listed in Table 1).

**Table 2:** Medical history of HP patients.

Events of HP patients, *n* (%)	Patients with HP (*n* = 22)
Previous EP	
0	15 (68.2)
1	3 (13.6)
2 and more	4 (18.2)
Previous abortion	
0	13 (59.1)
1	5 (22.7)
2 and more	4 (20.0)
Pelvic surgery history	
Tubal surgery	11 (50.0)
Laparoscopic adhesiolysis	4 (18.2)
Ovarian cyst excision	1 (4.5)
Appendectomy	4 (18.2)
None	5 (22.7)

The occurrence of HP had something to do with multiple transferred embryos and certain infertility factors. In our study, 13.6% (3/22) HP were transferred 3 embryos, while the counterpart in IUP was 0.08% (1/125). 86.4% (19/22) of HP had tubal infertility diagnosis, more than 4 times as the counterpart of intrauterine-only pregnancy (19.2%). And 31.8% of HP was diagnosed with pelvic adhesion, while only 8.0% of IUP had pelvic adhesion. We then assessed the relationship between HP and these 2 infertility factors and multiple transferred embryos. Tubal infertility and pelvic adhesion were both risk factors of HP after IVF. Pregnancy with tubal infertility factors had about 4 times higher risks of HP (OR 4.185, 95% CI 1.080–16.217); pelvic adhesion increased the risk of HP about 5 times (OR 5.552, 95% CI 1.677–18.382). And IVF with more than 2 embryos transferred had 23.253 times higher risks of HP (OR 23.253, 95% CI 1.804–299.767). In this study, we did not observe significant differences in age, previous ectopic pregnancies, previous abortions, previous live births, pelvic surgery history, type of IVF, stage of transferred embryos. Data was shown in Tables 3 and 4.

**Table 3:** Events of HP.

Factors*, n* (%)	HP (*n *= 22)	IUP (*n *= 125)	*P*
Age			.304
<30 y old	13 (59.1)	59 (47.2)	
≥30 y old	9 (40.9)	66 (52.8)	
Previous EP			.292
Yes	7 (31.8)	24 (19.2)	
No	15 (68.2)	101 (80.0)	
Previous abortions			.894
Yes	7 (31.8)	38 (30.4)	
No	15 (68.2)	87 (69.6)	
Previous live births			.266
Yes	3 (13.6)	6 (4.8)	
No	19 (86.4)	119 (95.2)	
Pelvic surgery history			.107
Yes	17 (77.3)	74 (59.2)	
No	5 (22.7)	51 (40.8)	
Type of IVF, *n* (%)			.203
Fresh cycle	17 (77.3)	112 (89.6)	
Frozen-thawed cycle	5 (22.7)	13 (10.4)	
Number of transferred embryos			.007
2	19 (86.4)	124 (99.2)	
3	3 (13.6)	1 (0.08)	
Stage of transferred embryos			.938
Day 3/cleavage stage	19 (86.4)	112 (89.6)	
Day 5/blastocyst stage	3 (13.6)	13 (10.4)	
Infertility factors			
Tubal infertility			<.001
Yes	19 (86.4)	24 (19.2)	
No	3 (13.6)	101 (80.8)	
Pelvic adhesion			.004
Yes	7 (31.8)	10 (8.0)	
No	15 (68.2)	115 (92.0)	

**Table 4:** Risk factors of HP.

Risk factors of HP	Odds risk (95% CI)
Tubal factor	4.185 (1.080–16.217)
Pelvic adhesion	5.552 (1.677–18.382)
More than 2 embryos transferred	23.253 (1.804–299.767)

HP surgery increased the risk of abortion. 27.8% of surgery-treated HP had abortion eventually, more than 2 times as the abortion rate of intrauterine-only pregnancy. In our study, 72.2% of surgery-treated HP had a live birth delivery, lower than the counterpart of intrauterine-only pregnancy (*P* = .042). Data was shown in Table 5.

**Table 5:** Pregnancy outcome after surgery.

Pregnancy outcome, % (*n*)	Surgery-treated HP (*n* = 18)	Intrauterine-only pregnancy (*n* = 3749)	*P*
Live birth	72.2% (13)	89.7% (3362)	.042
Abortion	27.8% (5)	10.3% (387)	

## 4 Discussion

HP is one of the ART's complications and rarely happens in spontaneous pregnancies. The typical symptoms are amenorrhea, vaginal bleeding, and abdominal pain coexistence with IUP. But some of HP have no symptoms and are diagnosed by routine TVS after ART procedures. Most of HP are diagnosed at first trimester, but some HP are diagnosed at second trimester.^[6,10]^ Because HP had risk of ectopic pregnancy rupture threatening life, it is vital to detect and diagnose HP as early as possible. Regular ultrasound examinations after ART may ensure detect and diagnosis HP at early stage. Fallopian, especially ampullary, is still the most common location of extrauterine lesion in HP. And the incidence of intestinal and cornual pregnancy in HP after ART counted at 18.2% and 13.6%, respectively. This increase might relate to IVF transplantation procedure. Transferred fertilized embryos moved to uterine corn or intestinal fallopian and then implanted. Besides HP occasionally happens in ovary, cervical, or spleen.^[7,11,12]^

Tubal infertility factors and pelvic adhesion increase the risk of HP after IVF. Tubal infertility is common in IVF population. In this study, the percentage of tubal infertility factors in HP was even higher, more than 4 times as that in IUP group (86.4% vs 19.2%, *P* < .001); and it was estimated that IVF with tubal infertility had more than 4 times higher risk of HP. This is consistent with other studies. Perkins et al^[3]^ suggested that tubal infertility factor increased risk of HP. And Liu et al^[13]^ reported that more HP had a history of hydrosalpinx. Xiao et al^[14]^ reported pelvic inflammation diseases was a risk factor of HP after IVF. Pelvic adhesion could be caused by pelvic inflammation diseases and surgery procedures.^[15]^ And in our study, we assessed pelvic adhesion in HP and concluded that it increased the risk of HP after IVF more than 5 times. This finding raised attention to reduce postoperative adhesion formation, in order to preserve infertility.

We also analyzed IVF-associated procedures, including the type of IVF cycles (fresh or frozen-thawed), the stage of transferred embryos, and number of transferred embryos. We found no significant difference between HP and IUP, except for number of transferred embryos. Studies showed that frozen-thawed cycles were associated with lower incidence of EP.^[16–18]^ However, in a retrospective study, Xiao et al^[14]^ compared fresh and frozen cycles in HP and found no statistically difference in the incidence of HP between these 2 groups. Blastocyst stage of transferred embryos used to be thought increased risk of EP than cleavage stage.^[19,20]^ However in later studies, it was proven that the stage of embryos had no association with the incidence of EP/HP.^[3,14,21]^ Multiple embryos transfer could increase risk of EP.^[3]^ In contrast, Xiao et al^[14]^ and Jeon et al^[21]^ suggested that 2 or 3 transferred embryos did not increase risk of HP. Consistent with Perkins,^[3]^ in our study we found that transferring of more than 2 embryos increased more than 20 times risk of HP. With the development of ART procedures, especially single-embryo transfer,^[22]^ limited number of transferred embryos to reduce multiple-pregnancy complications is the trend.

Laparoscopic surgery is widely used for HP diagnosis and treatment. It is considered as a safe approach, but its effect on pregnancy outcome of HP is uncertain. Soriano D et al.^[23]^ and Clayton HB et al.^[24]^ suggested laparoscopic treated HP had a higher risk of abortion. Similarly, we found surgery-treated HP had nearly 3 times rate of abortion, compared with clinical pregnancy in IVF.

The strength of our study is that we compared HP with clinical intrauterine-only pregnancy after IVF to identify risk factors and laparoscopic effect on pregnancy outcome. Our study is also subject to several limitations. First, we only collected data from our hospital and the HP sample was small. It may hide information because of the limited sample size and lead to false-negative results. Large sample study is required to further investigation. Next, our findings may not be generalized to the general population of female because they may have different risk factors than women undergoing IVF.

## 5 Conclusion

With widespread application of assisted reproductive technologies, the incidence of HP has risen. Our study found that tubal infertility and pelvic adhesion increased the risk of heterotopic pregnancy among IVF population. Abortion rate of heterotopic pregnancy increased after surgery treatment.

## Author contribution

PL and XQ: Project development, manuscript editing

RP: Project development, data analysis, manuscript writing

XZ: Protocol development

YL: Project development, DaSta collection, manuscript writing

**Conceptualization:** Xia Zhao, Ping Liu, Xiaorong Qi.

**Data curation:** Ruyu Pi, Yu Liu.

**Formal analysis:** Ruyu Pi.

**Investigation:** Yu Liu.

**Methodology:** Ruyu Pi, Yu Liu.

**Supervision:** Xia Zhao, Ping Liu, Xiaorong Qi.

**Writing – original draft:** Ruyu Pi, Yu Liu.

**Writing – review & editing:** Ping Liu, Xiaorong Qi.
